# 1,2-Dichloro-1,1,2,2-tetrafluoro­ethane: MAK Value Documentation, addendum – Translation of the German version from 2024

**DOI:** 10.34865/mb7614e11_1ad

**Published:** 2026-03-30

**Authors:** Andrea Hartwig, MAK Commission

**Affiliations:** 1 Institute of Applied Biosciences, Department of Food Chemistry and Toxicology, Karlsruhe Institute of Technology (KIT) Adenauerring 20a, Building 50.41 76131 Karlsruhe Germany; 2 Permanent Senate Commission for the Investigation of Health Hazards of Chemical Compounds in the Work Area, Deutsche Forschungsgemeinschaft, Kennedyallee 40, 53175 Bonn, Germany. Further information: Permanent Senate Commission for the Investigation of Health Hazards of Chemical Compounds in the Work Area | DFG

**Keywords:** 1,2-Dichloro-1,1,2,2-tetrafluoro­ethane, H-CFC, toxicity, ban of use, Luft, air

## Abstract

1,2-Dichloro-1,1,2,2-tetrafluoroethane [76-14-2] and other partly halogenated chloro­fluorocarbons (H-CFCs) are no longer approved in the European Union or in Germany. The derivation of the previous MAK value (maximum concentration at the workplace) of 1,2-dichloro-1,1,2,2-tetrafluoroethane by the German Senate Commission for the Investigation of Health Hazards of Chemical Compounds in the Work Area (MAK Commission) does not correspond to the current approach of the Commission. There are no new studies that would allow the MAK value to be revised. The Commission decided that a new evaluation is not of high priority. The MAK value and the other classifications are therefore suspended and the substance is listed in the Section II c of the List of MAK and BAT Values for substances no longer evaluated.

**Table:** 

**MAK value**	**See Section II c of the List of MAK and BAT Values**
**Peak limitation**	**–**
	
**Absorption through the skin**	**–**
**Sensitization**	**–**
**Carcinogenicity**	**–**
**Prenatal toxicity**	**–**
**Germ cell mutagenicity**	**–**
	
**BAT value**	**–**

1,2-Dichloro-1,1,2,2-tetrafluoroethane, also known as R114, freon 114 or cryofluorane, belongs to the class of partly halogenated chlorofluorocarbons (H-CFCs). It was used as a refrigerant, an aerosol propellant in sprays, a foaming agent in fire extinguishers, a blowing agent in cellular polymers, and as a solvent and cleaning agent in the electrical industry (FAN 2022).

Persistent compounds containing chlorine and bromine such as chlorofluorocarbons (CFCs) and halons can damage the stratospheric ozone layer. In 1987, the Montreal Protocol was therefore drawn up to protect the ozone layer. This was put in force in Europe and Germany by various regulations. The distribution and use of 1,2-dichloro-1,1,2,2-tetrafluoroethane has been banned in Europe since 1 January 2010. This is laid down by Regulation (EC) No 1005/2009 on substances that deplete the ozone layer (European Parliament and European Council 2009).

In 1958, a MAK value of 1000 ml/m^3^ was established for 1,2-dichloro-1,1,2,2-tetrafluoroethane, and documentation was published in 1972 (Henschler 1972, available in German only). In 2002, Peak Limitation Category II with an excursion factor of 8 was set (Greim 2002, available in German only).

The previous MAK value was not based on toxicological data, but rather on occupational hygiene considerations. The method used to derive the MAK value for 1,2-dichloro-1,1,2,2-tetrafluoroethane does not correspond to the current approach of the Commission. There are no new studies available that would allow the health hazard to be assessed. Re-evaluation of the substance is at present not of high priority. The MAK value, peak limitation category and classification in Pregnancy Risk Group D have therefore been suspended and 1,2-dichloro-1,1,2,2-tetrafluoroethane has been assigned to Section II c of the List of MAK and BAT Values.
